# Evaluating Sex-specific Differences in Abdominal Fat Volume and Proton Density Fat Fraction at MRI Using Automated nnU-Net–based Segmentation

**DOI:** 10.1148/ryai.230471

**Published:** 2024-05-29

**Authors:** Arun Somasundaram, Mingming Wu, Anna Reik, Selina Rupp, Jessie Han, Stella Naebauer, Daniela Junker, Lisa Patzelt, Meike Wiechert, Yu Zhao, Daniel Rueckert, Hans Hauner, Christina Holzapfel, Dimitrios C. Karampinos

**Affiliations:** From the Department of Diagnostic and Interventional Radiology, Klinikum rechts der Isar (A.S., M. Wu, S.R., J.H., S.N., D.J., L.P., D.C.K.), Institute of Nutritional Medicine, School of Medicine (A.R., M. Wiechert, H.H., C.H.), TUM School of Computation, Information, and Technology (Y.Z., D.R.), TUM School of Medicine and Health (D.R.), and Else Kröner Fresenius Center for Nutritional Medicine, School of Medicine (H.H.), Technical University of Munich, Ismaninger Str 22, 81675 Munich, Germany; Department of Computing, Imperial College London, London, UK (D.R.); Department of Nutritional, Food and Consumer Sciences, Fulda University of Applied Sciences, Fulda, Germany (C.H.); and Munich Institute of Biomedical Engineering and Munich Data Science Institute, Technical University of Munich, Garching, Germany (D.C.K.).

**Keywords:** Obesity, Chemical Shift–encoded MRI, Abdominal Fat Volume, Proton Density Fat Fraction, nnU-Net

## Abstract

Sex-specific abdominal organ volume and proton density fat fraction (PDFF) in people with obesity during a weight loss intervention was assessed with automated multiorgan segmentation of quantitative water-fat MRI. An nnU-Net architecture was employed for automatic segmentation of abdominal organs, including visceral and subcutaneous adipose tissue, liver, and psoas and erector spinae muscle, based on quantitative chemical shift-encoded MRI and using ground truth labels generated from participants of the Lifestyle Intervention (LION) study. Each organ’s volume and fat content were examined in 127 participants (73 female and 54 male participants; body mass index, 30–39.9 kg/m^2^) and in 81 (54 female and 32 male participants) of these participants after an 8-week formula-based low-calorie diet. Dice scores ranging from 0.91 to 0.97 were achieved for the automatic segmentation. PDFF was found to be lower in visceral adipose tissue compared with subcutaneous adipose tissue in both male and female participants. Before intervention, female participants exhibited higher PDFF in subcutaneous adipose tissue (90.6% vs 89.7%; *P* < .001) and lower PDFF in liver (8.6% vs 13.3%; *P* < .001) and visceral adipose tissue (76.4% vs 81.3%; *P* < .001) compared with male participants. This relation persisted after intervention. As a response to caloric restriction, male participants lost significantly more visceral adipose tissue volume (1.76 L vs 0.91 L; *P* < .001) and showed a higher decrease in subcutaneous adipose tissue PDFF (2.7% vs 1.5%; *P* < .001) than female participants. Automated body composition analysis on quantitative water-fat MRI data provides new insights for understanding sex-specific metabolic response to caloric restriction and weight loss in people with obesity.

**Keywords:** Obesity, Chemical Shift–encoded MRI, Abdominal Fat Volume, Proton Density Fat Fraction, nnU-Net

ClinicalTrials.gov registration no. NCT04023942

*Supplemental material is available for this article.*

Published under a CC BY 4.0 license.

**Summary**

Sex-specific differences in volume and fat fraction of abdominopelvic fat depots, liver, and two muscles were found in people with obesity before and after weight loss intervention using nnU-Net–based segmentation of chemical shift-encoded MRI.

**Key Points**

■ nnU-Net enabled robust segmentation of visceral adipose tissue, subcutaneous adipose tissue, liver, and the psoas and erector spinae muscle group of quantitative abdominal chemical shift–encoded MRI. Dice scores ranged from 0.91 to 0.97, enabling mean proton density fat fraction estimation per organ in addition to organ volume extraction.
■ Proton density fat fraction was overall higher in subcutaneous adipose tissue compared with visceral adipose tissue. It was found to be significantly higher in visceral adipose tissue (81.3% vs 76.4%; *P* < .001) and significantly lower in subcutaneous adipose tissue (89.7% vs 90.6%; *P* < .001) in male participants with obesity when compared with female participants with obesity.
■ During weight loss intervention, the change in the subcutaneous adipose tissue proton density fat fraction was higher in male participants (2.7% vs 1.5%; *P* < .001).

## Introduction

With the increasing prevalence of obesity (body mass index [calculated as weight in kilograms divided by height in meters squared] ≥ 30 kg/m^2^) (1), research investigating effective weight loss options has grown substantially (2,3). Studies using body composition analysis have shown that the risk for cardiovascular disease and type 2 diabetes is linked to specific body fat deposition patterns (4–7). Furthermore, a heterogeneous distribution of abdominal fat was reported between sexes and across age groups (8,9). To efficiently perform body composition profiling with MRI, accurate and automated segmentation tools are required.

Neural networks for abdominal adipose tissue segmentation have been based on T1-weighted images (10), turbo spin-echo images (11), as well as chemical shift–encoded MRI (CSE MRI) (11–13). Different network designs have been applied (14), including two-dimensional U-Net (12), a fusion of two-dimensional UNets (13), as well as a DC-Net (11). Recently, nnU-Net was used for segmentation of abdominal organs for volume estimation (10), which automatically sets the optimal training configurations and creates a specific pipeline of a U-Net–like network with its corresponding hyperparameters (15).

Previous MRI studies have primarily extracted organ volumes of visceral adipose tissue (VAT) and subcutaneous adipose tissue (SAT) (10–14). The use of CSE MRI (16) provides additional information on the fat content per voxel on quantitative proton density fat fraction (PDFF) maps, allowing the spatially resolved quantification of ectopic fat deposition in abdominal organs and muscle, as well as the detection of differences in adipose tissue hydration of VAT and SAT.

The present study aimed to develop an nnU-Net–based automated segmentation method based on water-fat images to quantify VAT, SAT, liver, psoas muscle, and erector spinae muscle volumes and extract their mean PDFF values. Additionally, this study aimed to apply the nnU-Net–based automated segmentation method in an abdominal CSE MRI dataset in people with obesity undergoing a weight loss intervention to assess body composition characteristics before and after intervention and their sex-related differences.

## Materials and Methods

### Study Sample

A total of 127 people with obesity participating in the Lifestyle Intervention (LION) study (17) underwent MRI from October 2019 until December 2021 (last follow-up). The study protocol was approved by the ethical committee of the Technical University of Munich (project no. 69/19S; ClinicalTrials.gov registration no. NCT04023942). Written informed consent was obtained from all participants. Inclusion and exclusion criteria are described in Reik et al (17).

The weight loss intervention consisted of an 8-week formula-based low-calorie diet of 800 kcal per day with an allowance of 200 g of nonstarchy vegetables. MRI was performed at baseline and after intervention. Due to COVID-19–related restrictions and dropouts, follow-up scans are available for 81 participants.

### MRI Measurements

Scans were performed with a 3-T scanner (Ingenia Elition X; Philips Healthcare). A six-echo three-dimensional (3D) multiecho gradient-echo sequence with bipolar gradient readouts was used (Table S1). Chemical shift encoding–based water-fat separation was performed with the scanner using a model employing a multipeak fat spectrum and a single T2* decay, which rendered water- and fat-separated images as well as PDFF and T2* maps. Participants were weighed in light clothing on a calibrated digital scale (Kern MPD 250 K100M; Kern & Sohn).

### 3D-UNet and nnU-Net Segmentation Algorithms

Manual segmentations were performed by medical students in their final year of training (J.H. and S.R.) who were supervised by two board-certified radiologists. Further details regarding generation of the ground truth labels are described in Appendix S1. The network architectures, 3D-UNet (18), and 3D-fullres nnU-Net (15) were employed for the segmentation algorithm and were implemented using PyTorch 1.7.0. Both networks were trained with fivefold cross-validation. 3D-UNet was trained for 180 epochs per fold, and the nnU-Net was trained for 500 epochs per fold. Available graphics cards included a 24 GB NVIDIA Quadro P6000 (NVIDIA) and a 12 GB NVIDIA Titan Xp (NVIDIA). Important Python libraries for the 3D-UNet include NumPy, Nibabel, and TensorBoard. Other libraries used in the ground truth generation and inference include SimpleITK, SciPy, scikit-learn, and NumPy. The FatSegNet (13) model was used with pretrained weights as part of the ground truth generation process. All programs were run on an Ubuntu 16.04 system (Canonical Foundation) with an Intel Core i7–6700 CPU (Intel) on a CuDNN version 8200 and CUDA 11.3. Our network results are available for download at *https://github.com/BMRRgroup/lion-abd-seg-nnunet* and *https://github.com/BMRRgroup/lion-abd-seg-3dunet*.

In total, 103 MRI datasets of 67 LION study participants with available ground truth labels from both baseline and follow-ups were split into 83 datasets for training and 20 datasets for testing. The 83 datasets used for training (among which 49 were female and 34 were male datasets) were split with a ratio of 4:1 for training and validation. When partitioning into training and test datasets, one participant’s images from the different time points were strictly put into either training or test groups. Training was performed either with a two-channel input using water- and fat-separated images or with a three-channel input adding background-removed T2* maps as a third channel. T2* maps were added because some anatomic structures, such as the vena cava, looked similar to the liver on the water-fat separated images but showed different contrast on the T2* maps. The network results were then applied to the unsegmented data to evaluate the cohort characteristics.

### Statistical Analysis

Statistical analysis of sex group differences was conducted in Python (version 3.8; Python Software Foundation). The null hypothesis represented the outcome that there was no difference between sexes. The Shapiro-Wilk test was applied to test for normal distribution. A *t* test was used in case of normal distribution, and the Mann-Whitney *U* test was applied otherwise. *P* values less than .05 were considered to indicate a statistically significant difference. No adjustment for multiple testing was made. An interrater agreement of the ground truth label generation was assessed with Bland-Altman plots. Dice scores were computed to assess the performance of different neural networks.

## Results

**Figure 1:**
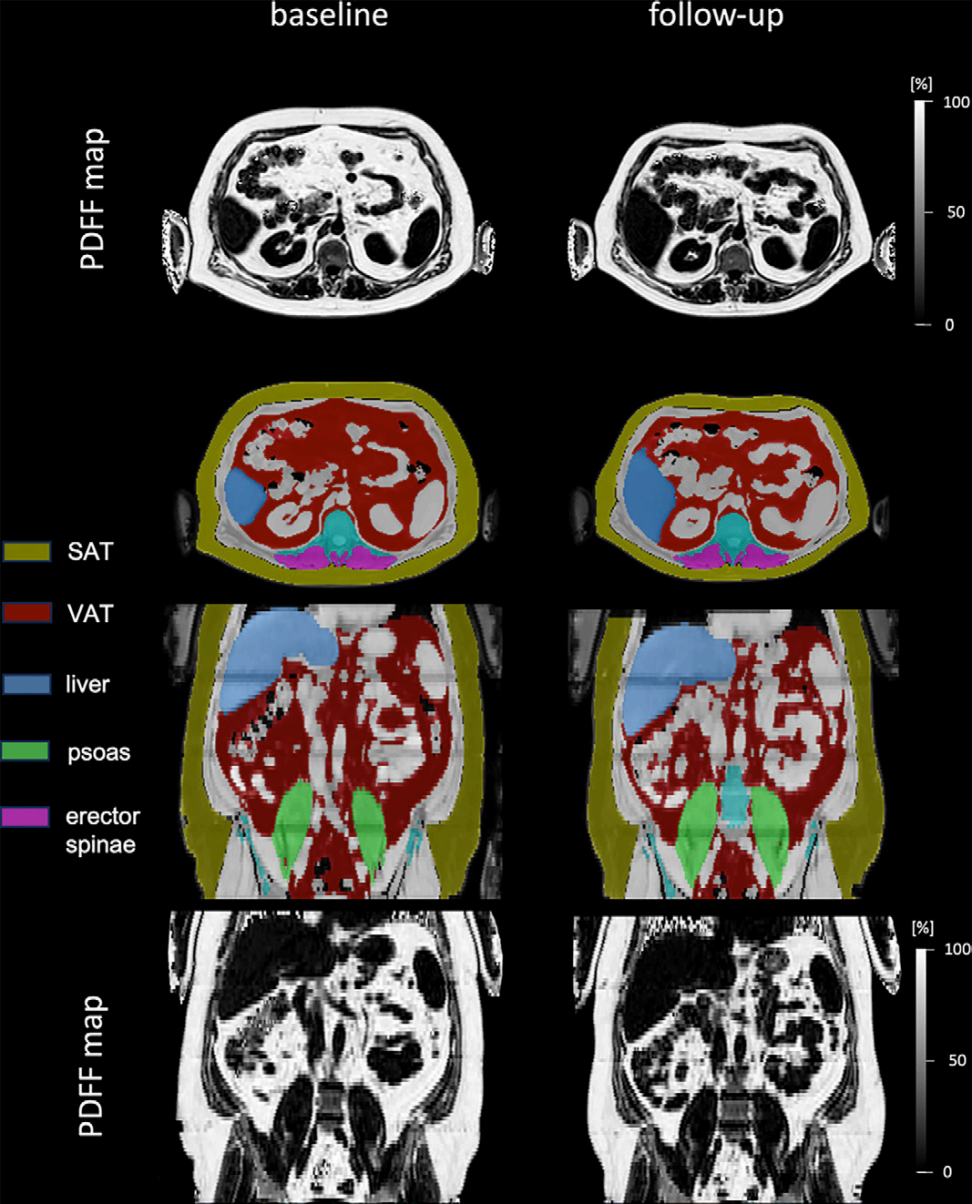
Axial and coronal view of MRI-based proton density fat fraction (PDFF) maps and corresponding organ segmentations in a male participant (age at baseline, 43 years; body mass index [BMI; calculated as weight in kilograms divided by height in meters squared] at baseline, 38.8 kg/m^2^) before and after the weight loss intervention. The participant lost 14.8 kg (from 147.5 kg to 132.7 kg) and decreased his BMI by 3.9 kg/m^2^. The decrease in visceral adipose tissue (VAT) volume from 11.55 L to 8.32 L can be detected visually. SAT = subcutaneous adipose tissue.

A fully automated multiorgan segmentation of the abdominal and pelvic region for body composition analysis was employed based on CSE MRI. Selected characteristics of the study cohort (73 male and 54 female participants) at baseline and after weight loss intervention (54 male and 32 female participants) are shown in the Table. Figure 1 shows an example of organ segmentation in a male participant before and after weight loss intervention.

**Table:**
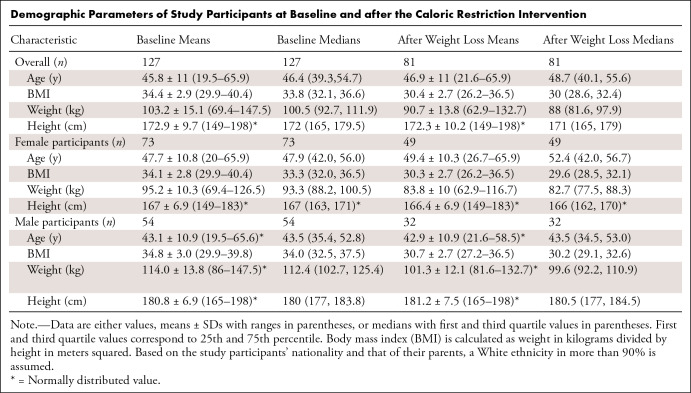
Demographic Parameters of Study Participants at Baseline and after the Caloric Restriction Intervention

### 3D-UNet and nnU-Net Performance Comparison

**Figure 2:**
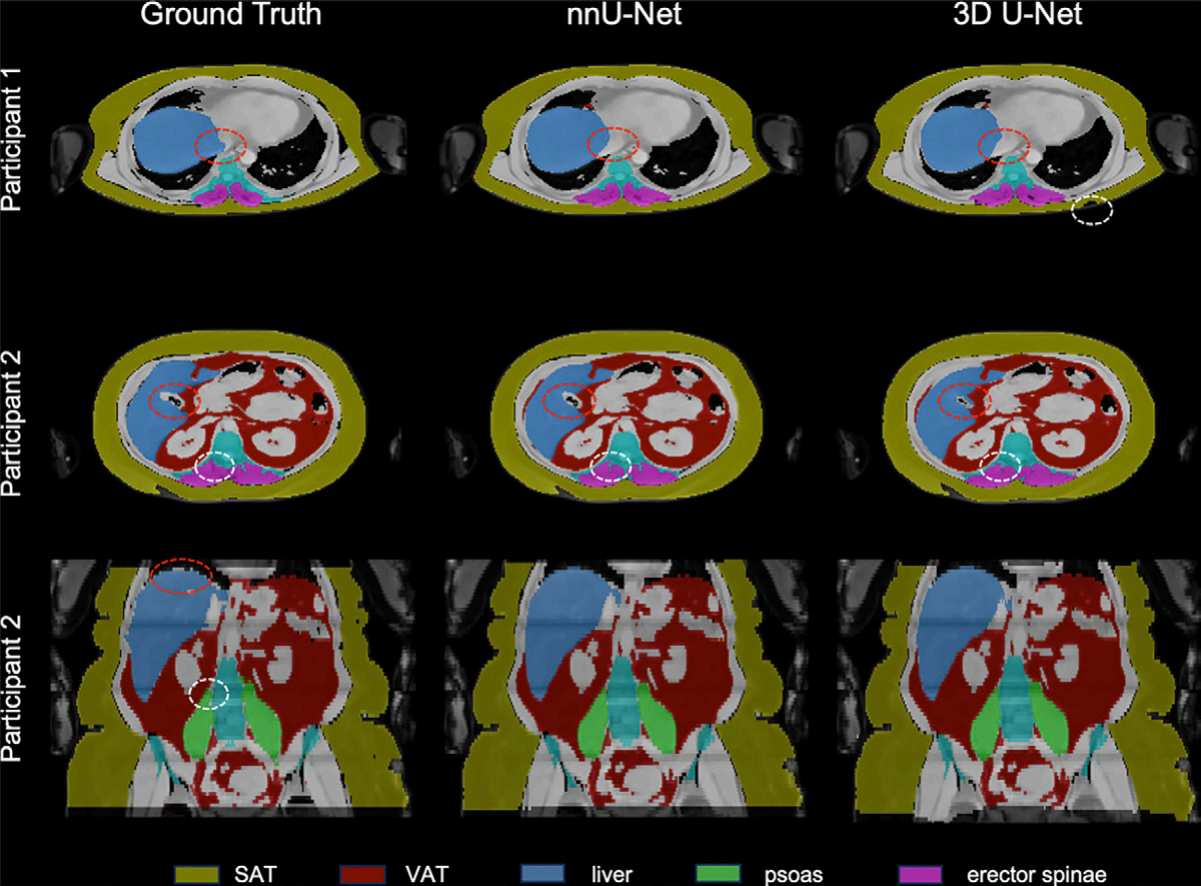
Example segmentations compare ground truth and nnU-Net and three-dimensional (3D) U-Net neural network predictions for two participants. In participant 1 (first row), the vena cava was correctly spared in both network predictions (shown in red circles), while the human misinterpreted the vessel as part of the liver. The white circle shows a subcutaneous adipose tissue (SAT) region of interest that was missed by the 3D U-Net. In the axial view of participant 2 (second row), the red circle shows the gallbladder, which was in part wrongly classified as liver by the 3D U-Net. The white circle shows vertebral bone parts that were delineated as part of the erector spinae by the manual annotator but were correctly spared in the neural network predictions. The coronal view of participant 2 (third row) shows smooth borders for the neural network solutions compared with the manual segmentation (red circle) or the psoas muscle (white circle). VAT = visceral adipose tissue.

A slight improvement of the Dice score was observed for all organs when using background-removed T2* maps as an additional input channel to the 3D-UNet (Table S2). Thus, the nnU-Net was trained with all three channels. The nnU-Net showed a better performance than the 3D-UNet both by visual inspection of the segmentation outcome (Fig 2) and by using the Dice score (Table S2). For the erector spinae segmentation, the nnU-Net Dice score was higher when compared with the interrater evaluation (Table S2). Bland-Altman plots illustrating the interrater evaluation and both network results compared with ground truth labels for each organ’s volume and mean PDFF are presented in Figure S1. Using the same hardware, the 3D-UNet three-channel training with 180 epochs required 12 hours per fold, whereas the nnU-Net with 500 epochs required 67 hours per fold.

### Organ Volumes and Mean PDFF at Baseline and after Weight Loss Intervention

**Figure 3:**
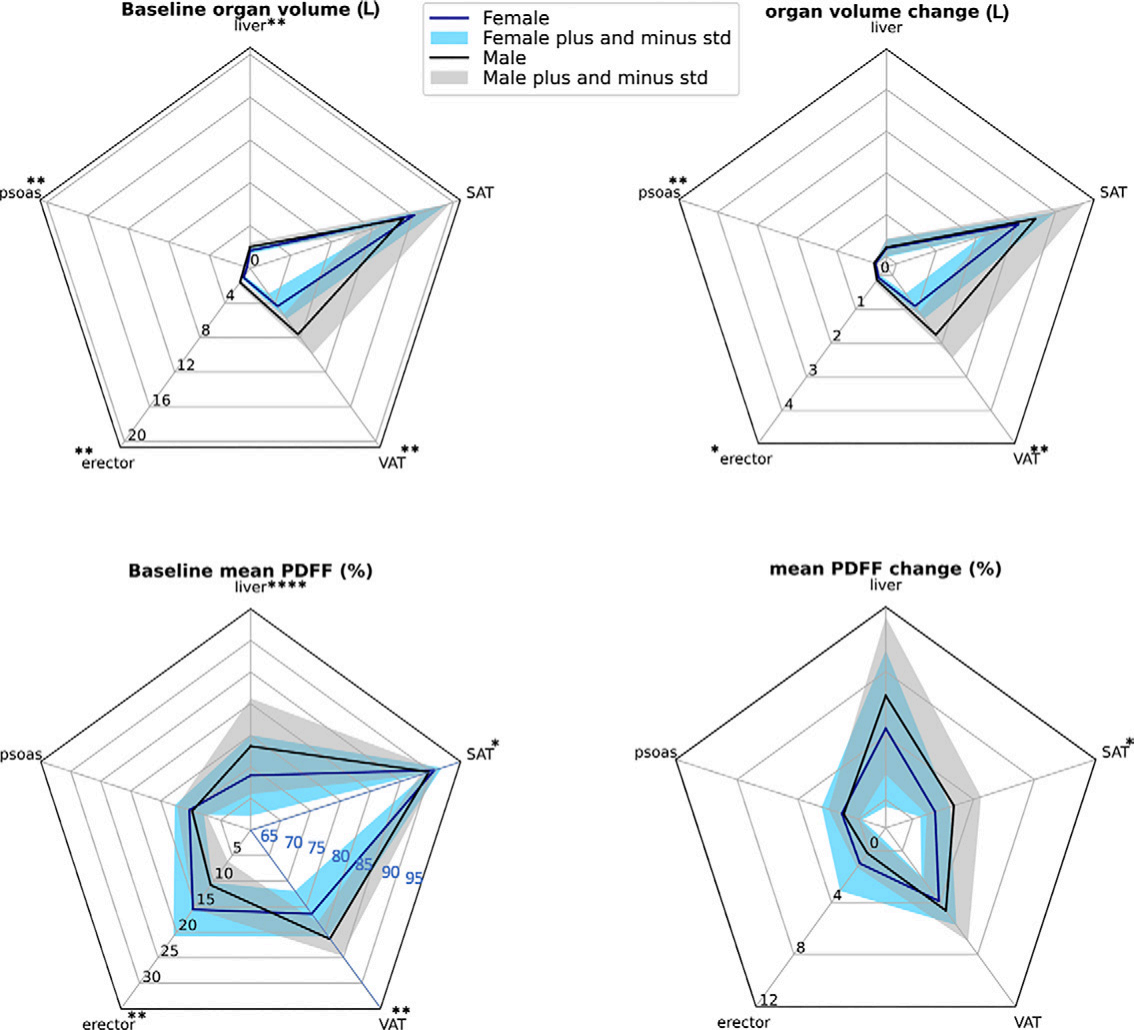
Radar plots display mean organ volume and mean organ proton density fat fraction (PDFF) at baseline and their changes after weight loss intervention, stratified by sex. Sex-related statistical differences are indicated (Table S3). The radar plots of the first row show that subcutaneous adipose tissue (SAT) volume loss was the largest contributor to weight loss. All volumes except for SAT volume were larger in male participants at baseline. Organ volume change in male participants was significantly larger in visceral adipose tissue (VAT) and the psoas and erector spinae muscle. The radar plots of the second row show that baseline liver and VAT PDFF were higher in male participants, and the erector muscle and SAT PDFF exhibited significantly higher values in female participants (SAT and VAT PDFF at baseline were scaled with an offset of 60% for better visualization). PDFF change was significantly higher in male participants for only SAT. std = standard deviation. * = *P* < .001, ** = *P* < .0001.

Organ-specific volumes and PDFFs were estimated before and after an 8-week formula-based weight loss intervention. The average weight loss after 8 weeks of a low-calorie diet was 10.7 kg, with male participants showing a significantly greater weight loss than female participants (12.2 kg vs 9.8 kg; *P* < .001). The change in organ volume and mean PDFF is plotted for all 81 participants in Figure S2. Abdominal adipose tissue volumes and intraorgan ectopic fat deposition depended on sex. All organ volumes at baseline were significantly larger in male participants with obesity (*P* < .001) except for SAT volume (Fig 3; Table S3). Absolute VAT volume change after weight loss intervention was significantly higher in male than female participants (1.76 L vs 0.91 L; *P* < .001). The volume change in SAT was the highest contributor to weight loss for both male and female participants. Significant differences in organ volume change between sexes was observed in the psoas muscle, erector muscle, and VAT (all *P* < .001; Table S3), whereas no evidence of a difference between sexes was found for SAT and liver volume change. A liver volume reduction of about 200 mL was observed in both sexes. In relation to the baseline SAT and VAT volume, male participants lost proportionally more SAT and VAT compared with female participants after the intervention (Fig S3; Table S3).

Mean liver PDFF at baseline was significantly higher in male participants (13.3% vs 8.6%; *P* < .001), and the observed change in mean liver PDFF was also higher in male participants but did not reach statistical significance (*P* = .053). The baseline mean PDFF in the erector spinae was higher in female participants (15.6% vs 10.8%; *P* < .001). Even though mean SAT PDFF was significantly higher in female participants at baseline (90.6% vs 89.7%; *P* < .001), the change of SAT PDFF was higher in male participants (2.7% vs 1.5%; *P* < .001). Moreover, the mean PDFF was significantly higher in VAT for male participants (81.3% vs 76.4%; *P* < .001). No evidence of a sex-specific difference for the PDFF was detected in the psoas muscle at baseline and after the low-calorie diet.

## Discussion

In addition to the quantification of adipose tissue volumes, the present automated segmentation enabled the quantification of ectopic lipids in the liver and skeletal muscle as well as the fat fraction within the adipose tissue, which relies on the use of a quantitative water-fat imaging acquisition (16). The nnU-Net outperformed the 3D-UNet–based segmentation in terms of Dice score as well as visual inspection of organ delineation.

The application of the segmentation algorithm provided a comprehensive analysis of sex-related body composition characteristics for the LION participants with obesity before and after weight loss. The results reported here showed specific patterns and temporal changes in adipose tissue volumes, as well as skeletal muscle and liver ectopic fat deposition. Regarding adipose tissue, a higher VAT volume was associated with higher VAT PDFF, similar to previous literature (19). Higher baseline VAT volume and higher absolute changes in VAT volume during weight loss were observed in male participants in comparison to female participants, which has also been discussed previously (8). Significantly higher liver PDFF values were found in male participants of the present study (20), while no evidence of a sex-related difference in liver PDFF change with diet was found. Comparatively smaller adipocytes were previously observed in female individuals (21). However, another study of female individuals with obesity revealed that SAT was subject to both hyperplasia and hypertrophy, whereas VAT exhibited primarily hypertrophic cells (22). The greater PDFF change in SAT with the low-calorie diet in male participants may indicate higher adipose tissue hydration as a response to the caloric restriction in a tissue with predominantly hypertrophic adipocytes. Interestingly, a recent study found that the increase in water content in SAT after rapid weight loss correlated with an increase in insulin sensitivity (23). Regarding skeletal muscle fat, fatty infiltration in the erector muscle was higher in female participants, as previously reported in a healthy cohort (24).

Our study had important limitations. The statistical tests did not consider multiple hypothesis testing or confounders such as body size. Reproducible organ delineation depends on ground truth definition and is limited by partial volume effects in organs with many air-tissue interfaces (VAT) or big gradients of PDFF at the tissue border (muscle). As the erector spinae muscle may be infiltrated and surrounded by fat voxels in a person with obesity, a considerable variability, especially in the mean PDFF estimation, was observed for both the interrater evaluation and the network predicted labels due to a challenging muscle border definition. Despite being trained on data from people with a body mass index range of 26–40 kg/m^2^, the network performed well on images of a person with a body mass index of 23.1 kg/m^2^ at the 12-month follow-up scan (Fig S4). The segmentation performance with imaging data outside our institution remains to be examined, as whole abdominal six-echo CSE MR images were not publicly available yet. Limited performance may be expected on images with different MR image contrast, such as T1-weighted two-point Dixon water-fat images, as shown in the supplemental materials. Further investigation of the relation between individual abdominal fat distribution and cardiometabolic health is needed and will profit from multiorgan segmentation beyond VAT and SAT volume estimation. With this new knowledge, individualized risk profiles and targeted prevention programs can be generated based on body composition analysis. The metabolic processes behind a caloric restriction can also be better understood with abdominal organ volume and PDFF estimation.

In conclusion, automated body composition and abdominal ectopic lipid quantification is feasible when the proposed deep learning–based technique is applied for multiorgan segmentation of quantitative water-fat MRI data. The automated segmentation of SAT, VAT, the whole liver, and two distinct muscle groups provided new insights for a better understanding of sex-dependent abdominal fat distribution and changes in response to a weight loss intervention in people with obesity.

## References

[1] Ward ZJ , Bleich SN , Cradock AL , et al . Projected U.S. State-Level Prevalence of Adult Obesity and Severe Obesity . N Engl J Med 2019 ; 381 ( 25 ): 2440 – 2450 . 31851800 10.1056/NEJMsa1909301

[2] Vogt LJ , Steveling A , Meffert PJ , et al . Magnetic Resonance Imaging of Changes in Abdominal Compartments in Obese Diabetics during a Low-Calorie Weight-Loss Program . PLoS One 2016 ; 11 ( 4 ): e0153595 . 27110719 10.1371/journal.pone.0153595 PMC4844151

[3] Ross R , Bradshaw AJ . The future of obesity reduction: beyond weight loss . Nat Rev Endocrinol 2009 ; 5 ( 6 ): 319 – 325 . 19421242 10.1038/nrendo.2009.78

[4] Linge J , Borga M , West J , et al . Body Composition Profiling in the UK Biobank Imaging Study . Obesity (Silver Spring) 2018 ; 26 ( 11 ): 1785 – 1795 . 29785727 10.1002/oby.22210 PMC6220857

[5] Agrawal S , Klarqvist MDR , Diamant N , et al . BMI-adjusted adipose tissue volumes exhibit depot-specific and divergent associations with cardiometabolic diseases . Nat Commun 2023 ; 14 ( 1 ): 266 . 36650173 10.1038/s41467-022-35704-5 PMC9844175

[6] Shungin D , Winkler TW , Croteau-Chonka DC , et al . New genetic loci link adipose and insulin biology to body fat distribution . Nature 2015 ; 518 ( 7538 ): 187 – 196 . 25673412 10.1038/nature14132 PMC4338562

[7] Wagner R , Heni M , Tabák AG , et al . Pathophysiology-based subphenotyping of individuals at elevated risk for type 2 diabetes . Nat Med 2021 ; 27 ( 1 ): 49 – 57 . 33398163 10.1038/s41591-020-1116-9

[8] Palmer BF , Clegg DJ . The sexual dimorphism of obesity . Mol Cell Endocrinol 2015 ; 402 : 113 – 119 . 25578600 10.1016/j.mce.2014.11.029 PMC4326001

[9] Zeng Q , Wang L , Dong S , et al . CT-derived abdominal adiposity: Distributions and better predictive ability than BMI in a nationwide study of 59,429 adults in China . Metabolism 2021 ; 115 : 154456 . 33259834 10.1016/j.metabol.2020.154456

[10] Kart T , Fischer M , Küstner T , et al . Deep Learning-Based Automated Abdominal Organ Segmentation in the UK Biobank and German National Cohort Magnetic Resonance Imaging Studies . Invest Radiol 2021 ; 56 ( 6 ): 401 – 408 . 33930003 10.1097/RLI.0000000000000755

[11] Küstner T , Hepp T , Fischer M , et al . Fully Automated and Standardized Segmentation of Adipose Tissue Compartments via Deep Learning in 3D Whole-Body MRI of Epidemiologic Cohort Studies . Radiol Artif Intell 2020 ; 2 ( 6 ): e200010 . 33937847 10.1148/ryai.2020200010 PMC8082356

[12] Wang Z , Cheng C , Peng H , et al . Automatic segmentation of whole-body adipose tissue from magnetic resonance fat fraction images based on machine learning . MAGMA 2022 ; 35 ( 2 ): 193 – 203 . 34524564 10.1007/s10334-021-00958-5

[13] Estrada S , Lu R , Conjeti S , et al . FatSegNet: A fully automated deep learning pipeline for adipose tissue segmentation on abdominal dixon MRI . Magn Reson Med 2020 ; 83 ( 4 ): 1471 – 1483 . 31631409 10.1002/mrm.28022 PMC6949410

[14] Greco F , Mallio CA . Artificial intelligence and abdominal adipose tissue analysis: a literature review . Quant Imaging Med Surg 2021 ; 11 ( 10 ): 4461 – 4474 . 34603998 10.21037/qims-21-370 PMC8408793

[15] Isensee F , Jaeger PF , Kohl SAA , Petersen J , Maier-Hein KH . nnU-Net: a self-configuring method for deep learning-based biomedical image segmentation . Nat Methods 2021 ; 18 ( 2 ): 203 – 211 . 33288961 10.1038/s41592-020-01008-z

[16] Reeder SB , Hu HH , Sirlin CB . Proton density fat-fraction: a standardized MR-based biomarker of tissue fat concentration . J Magn eason Imaging 2012 ; 36 ( 5 ): 1011 – 1014 . 10.1002/jmri.23741 PMC4779595 22777847

[17] Reik A , Holzapfel C . Randomized Controlled Lifestyle Intervention (LION) Study for Weight Loss and Maintenance in Adults With Obesity-Design and Methods . Front Nutr 2020 ; 7 : 586985 . 33240920 10.3389/fnut.2020.586985 PMC7683381

[18] Çiçek Ö , Abdulkadir A , Lienkamp SS , Brox T , Ronneberger O . 3D U-Net: Learning Dense Volumetric Segmentation from Sparse Annotation . In: Ourselin S , Joskowicz L , Sabuncu MR , Unal G , Wells W , eds. Medical Image Computing and Computer-Assisted Intervention – MICCAI 2016. MICCAI 2016. Lecture Notes in Computer Science , vol 9901 . Springer , 2016 ; 424 – 432 .

[19] Franz D , Weidlich D , Freitag F , et al . Association of proton density fat fraction in adipose tissue with imaging-based and anthropometric obesity markers in adults . Int J Obes 2018 ; 42 ( 2 ): 175 – 182 . 10.1038/ijo.2017.194 PMC5737837 28894290

[20] Bertolotti M , Lonardo A , Mussi C , et al . Nonalcoholic fatty liver disease and aging: epidemiology to management . World J Gastroenterol 2014 ; 20 ( 39 ): 14185 – 14204 . 25339806 10.3748/wjg.v20.i39.14185 PMC4202348

[21] Tchoukalova YD , Koutsari C , Karpyak MV , Votruba SB , Wendland E , Jensen MD . Subcutaneous adipocyte size and body fat distribution . Am J Clin Nutr 2008 ; 87 ( 1 ): 56 – 63 . 18175737 10.1093/ajcn/87.1.56

[22] Drolet R , Richard C , Sniderman AD , et al . Hypertrophy and hyperplasia of abdominal adipose tissues in women . Int J Obes 2008 ; 32 ( 2 ): 283 – 291 . 10.1038/sj.ijo.0803708 17726433

[23] Laaksonen DE , Nuutinen J , Lahtinen T , Rissanen A , Niskanen LK . Changes in abdominal subcutaneous fat water content with rapid weight loss and long-term weight maintenance in abdominally obese men and women . Int J Obes Relat Metab Disord 2003 ; 27 ( 6 ): 677 – 683 . 12833111 10.1038/sj.ijo.0802296

[24] Crawford RJ , Filli L , Elliott JM , et al . Age-and Level-Dependence of Fatty Infiltration in Lumbar Paravertebral Muscles of Healthy Volunteers . AJNR Am J Neuroradiol 2016 ; 37 ( 4 ): 742 – 748 . 26635285 10.3174/ajnr.A4596 PMC7960169

